# Mutual Relationship Between Sleep Disorders, Quality of Life and Psychosocial Aspects in Patients With Psoriasis

**DOI:** 10.3389/fpsyt.2021.674460

**Published:** 2021-07-06

**Authors:** Julia Nowowiejska, Anna Baran, Iwona Flisiak

**Affiliations:** Department of Dermatology and Venereology, Medical University of Bialystok, Bialystok, Poland

**Keywords:** psoriasis, sleep, sleep disorders, life quality, productivity

## Abstract

Psoriasis is a chronic, autoimmune skin disease affecting about 2–4% of the worldwide population. It is now perceived as a systemic disease because of the complex pathogenesis and multiple comorbidities. It leads to decreased quality of life and productivity of patients. Nowadays, sleep disorders are investigated as well in relation to psoriasis as another possible comorbidity. This review focuses on possible negative effects of sleep deprivation, decreased quality of life, and psychosocial status in patients with psoriasis and highlights their mutual, complex relationship of divergent consequences. The relationship between sleep disorders and psychosocial status in patients with psoriasis is bidirectional and resembles a vicious circle, one abnormality triggering the other. Sleep disorders additionally increase the risk of metabolic and psychiatric diseases in psoriatic patients who are already at increased risk of developing such disorders. There should be measures taken to screen patients with psoriasis for sleep disorders in order to diagnose early and treat.

## Introduction

Sleep is an essential physiological process that is characterized by altered consciousness and changes in mind and body organ functions in a cyclic manner (1, 2). It is estimated that an average person spends about one third of their lifetime sleeping (3). The role of sleep in human health maintenance is enormous because it is associated with cognitive function, memory and mood, immunity, and endocrine and cardiovascular function as well as musculoskeletal system recovery (1, 4).

**Figure 1:**
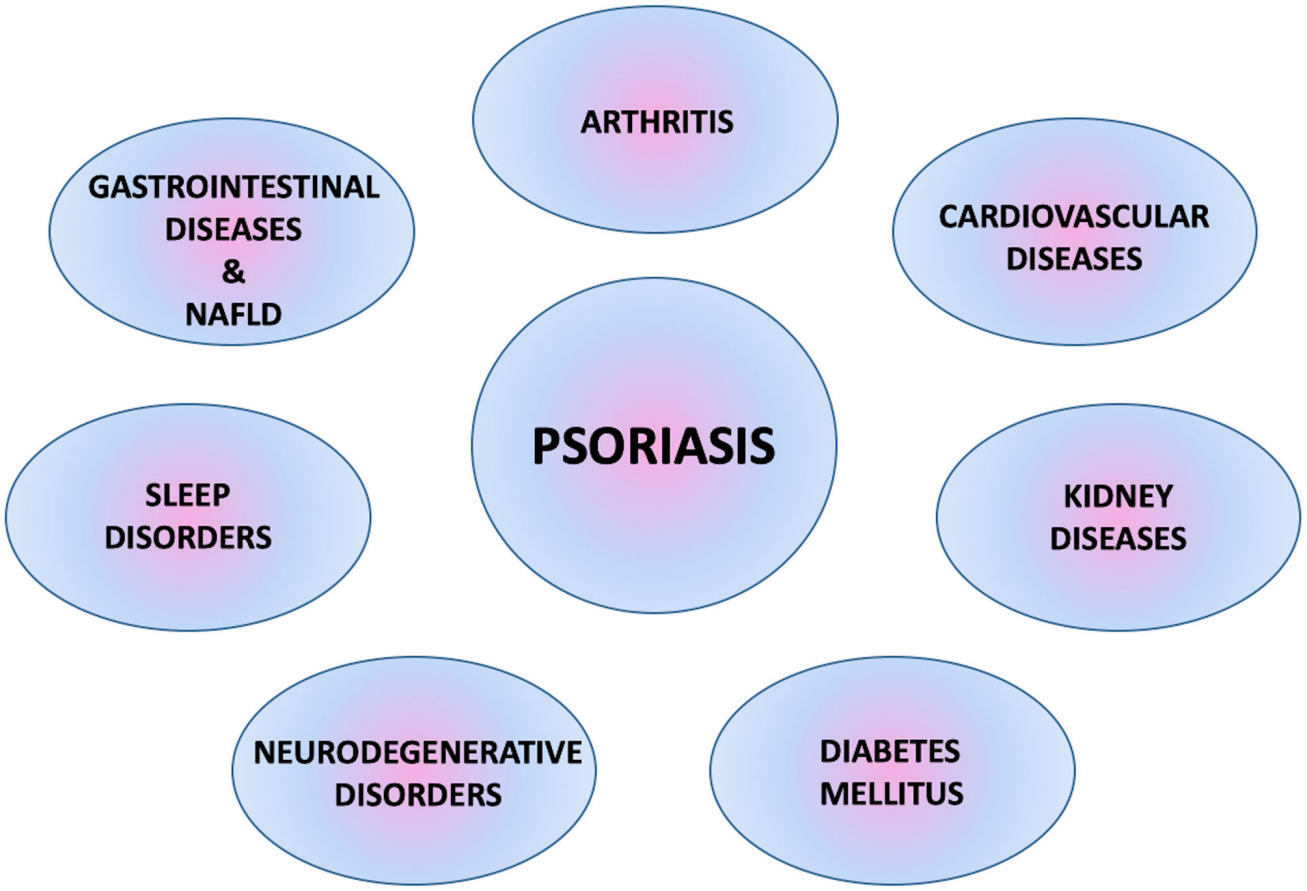
Multiple comorbidities associated with psoriasis.

Psoriasis is one of the most common dermatological diseases in clinical practice. Nowadays, it is treated not only as a skin-related problem, but more as a disorder characterized by autoimmune and inflammatory processes affecting the body's organs and accompanied by multiple comorbidities (Figure 1) (5, 6).

Sleep and quality of life, along with psychosocial aspects in patients with psoriasis are bonded together through a network of mutual dependencies, one affecting the other bidirectionally. This review aims to show numerous possible interactions between these issues in this particular group of patients and highlight the problem of multifactorial influence and reasons for sleep and psychosocial disturbances. Available literature data suggest more frequent occurrence of insomnia, obstructive sleep apnea syndrome (OSAS), and restless leg syndrome (RLS) in patients with psoriasis along with decreased quality of life and psychosocial matters although there appear some inconsistencies that require further research (7, 8). Interestingly, sleep disorders (SD) seem to be a crucial and underestimated comorbidity in psoriasis, acting as both a mediator and effect.

## Bidirectional Relationship Between Skin and Sleep

As the biggest human body organ, skin also affects sleep. It plays a role in thermoregulation and control of body core temperature, which influences the sleeping process (9, 10). Moreover, a pathologic situation caused by skin disease occurrence has a major impact on this matter. Dermatoses may present with symptoms of pruritus, pain, burning sensations, or in the case of underlying tissues or joints being involved, with pain or inability to move (11). The mentioned subjective symptoms can cause difficulty with falling asleep, awakening during the course of sleep, or getting up too early and unrested (12). Skin diseases frequent in daily medical practice, which can cause such symptoms are, for instance, psoriasis and psoriatic arthritis, atopic dermatitis, lichen planus, or chronic urticaria (11, 13). SD in such morbidities has already been investigated, bringing this matter to physicians' attention (11, 13, 14). They are elusive because they are not easy to notice or describe by patients and not visible or instantly recognized by doctors at the appointment. What is essential is that, skin diseases, especially chronic ones, often coexist with depression or anxiety, which are known to cause sleep disturbances (15).

Insufficient sleep also influences skin. It is reported that lack of sleep causes faster skin aging and even defects in skin barrier functions with inadequate response to exogenic factors (9). Research shows that individuals affected with sleep loss present increased transepidermal water loss, uneven skin pigmentation, fine wrinkling, and more intense skin laxity along with subcutaneous fat reduction (9).

## Negative Consequences of Sleep Deprivation

However intangible, sleep deprivation is a real medical problem. It is even regarded as a public health epidemic according to the U.S. Centers for Disease Control (9, 16). Actions resulting in sleep deprivation are some of the most severe kinds of psychological torture known to humanity (17, 18). The first, most obvious result of sleep deprivation is the decreased quality of life of affected individuals (9). Insufficient sleep and the feeling of tiredness lead to low mood or, in severe and chronic cases, even psychological disorders (12, 19). It is established that sleep loss is associated with increased risk of depression, anxiety, and suicidal tendencies (12). Another problem is increased fatigue, sleepiness, and decreased concentration during daytime. It is related to low energy to perform daily activities, an inability to fulfill one's duties, problems with decision-making processes, and an increased rate of mistakes (20). It can also increase the probability of a car accident and death (21). Fatigue and cognitive impairment due to sleep deprivation prevent individuals from working or learning to a sufficient extent (12, 22, 23). People who suffer from such disturbances are reported to be less productive employees as a result of which they may lose their job and consequently have their economic status decreased (23, 24). Some research shows that insufficient sleep generates measurable costs (24). One example can be the report on costs that are incurred by five big Organization for Economic Cooperation and Development countries due to sleep deprivation. They were estimated to be 680 billion dollars of economic output a year and to continue to rise over time (23). Inability to perform everyday activities may also involve physical activity. Nowadays, rushing, a sedentary lifestyle, and fatigue make it hard to perform high-intensity workouts, which results in obesity and its comorbidities (25). Sleep deprivation also makes social interactions difficult. It is shown that insufficient sleep negatively affects the ability of concentration in contact with other people, understanding facial expressions (and, therefore, emotions) and adequate social decision-making processes (24). Moreover, not only does chronic fatigue and sleepiness prevent individuals from socializing and bonding with one another (26), it also can cause a lower sex drive and problems with sexual life (23). Sleep loss and insufficient rest lead to increased stress and even anxiety development (27). Prolonged sleep loss may alter neurotransmission processes and neuroendocrine reactivity similarly as observed in depression, which could provide further evidence that chronic stress experience and sleep deprivation may trigger mood disorders (28).

Besides psychological consequences, sleep deprivation can have somatic ones. Sleep loss is bidirectionally associated with function of the hypothalamic–pituitary–adrenal (HPA) axis. On the one hand, increased activation of the HPA axis results in sleeplessness symptoms; on the other hand, sleep disturbances act due to promoting the activity of the same axis (19). It results in increased secretion of cortisol and pro-inflammatory cytokines, e.g., interleukin 6 (IL-6) and tumor necrosis factor alpha, which negatively affect immune and autonomic nervous system function, also leading to impairment of cognitive function and change in pain perception (4, 28). Numerous studies reveal decreased serum adiponectin levels or increased ghrelin and leptin levels in individuals affected by sleep deprivation (29, 30). Such imbalance between the latter adipokines is suspected to be linked to increased hunger and, therefore, calorie intake (29). These findings suggest increased risk of obesity and impaired glucose metabolism due to insufficient sleep (30). Sleep loss also affects blood vessel endothelium function, which results in insufficient vasodilatation. An additional negative effect is the elevated sympathetic activity leading to vasoconstriction. It is clearly documented that such individuals present an increased risk of cardiovascular and metabolic diseases, e.g., arterial hypertension, diabetes mellitus, or obesity, and therefore greater probability of death due to cardiovascular incidences (9). Circadian rhythm also affects secretion of a few sex hormones, which influence human reproduction (31). The role of insufficient sleep duration or interrupted sleep course is previously reported as a factor contributing to susceptibilities to infectious diseases (32). Increased risk of neoplasms due to sleep disturbances is also mentioned (28).

## Negative Effects of Decreased Quality of Life and Psychoemotional and Socioeconomic Burden on Sleep Quality

Many factors can have a significant impact on quality of life, among them are health condition and psychoemotional and socioeconomic status. Dissatisfaction within any of these areas can contribute to development of sleep disturbances.

Low income, inability to provide for one's own family, or even financial troubles and debts cause major stress and psychological burden. Other described factors are progressive aging and insufficient access to health care. These can undoubtedly lead to SD (2, 28). It must be also taken into account that exposure to stress or the abovementioned deterioration in socioeconomic status increase the possibility of addiction to such substances, such as alcohol, cigarettes, or drugs (33). Alcohol and cigarettes are known to increase risk of OSAS (34–36). Moreover, psychostimulants can cause sleeplessness (37). Low socioeconomic status may also lead to social deterioration (38), lower physical activity level (39), and unbalanced diet (40).

People who have experienced objective social isolation are reported to suffer from SD as well as depression and fatigue more often (41). It is observed that subjective prolonged sleep latency is associated with exposure to psychosocial triggering factors (these are described as, e.g., trouble at work or in family) the exact same day. On the other hand, researchers have found shortened sleep duration and worse sleep quality to be influenced by the exposure to such factors the following day (20, 42). Studies show that the feeling of social isolation is associated with increased secretion of pro-inflammatory cytokines as well as pro-inflammatory gene expression and alterations in hormone signaling, similar to what we observe after sleep deprivation (4, 28, 43). Research has also revealed that social isolation may independently trigger health-damaging behaviors, for instance, smoking and alcohol abuse, which are mentioned above as contributing to sleep deprivation (38).

To sum up, it is reported that psychosocial stress, inappropriate dietary habits, and insufficient physical activity are common factors responsible for sleep deprivation in contemporarily living people (13, 38).

## The Influence of Psoriasis on Sleep Course

### The Influence of Psoriasis on Insomnia

**Figure 2:**
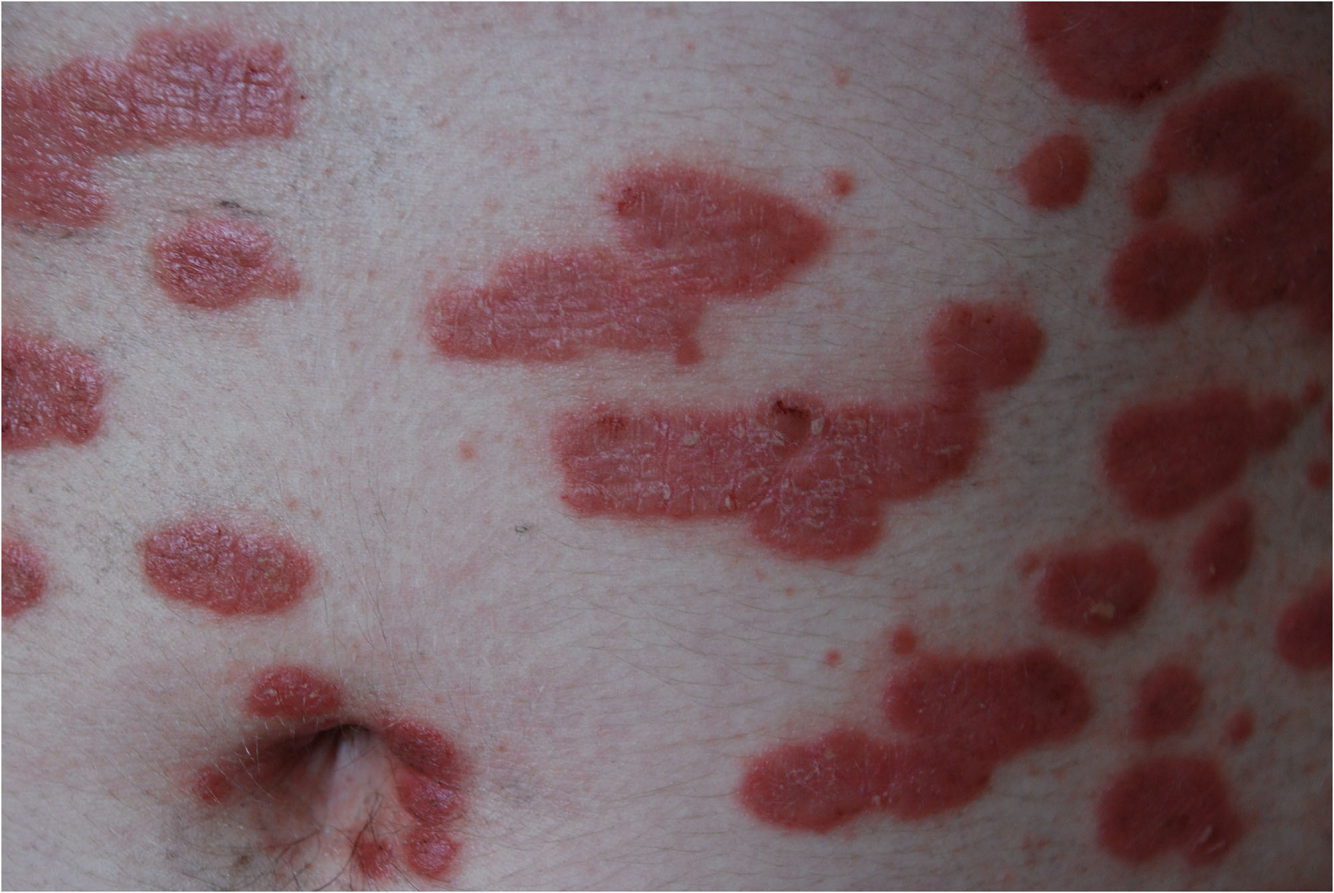
Erythematous-infiltrative psoriatic lesions on the trunk (from the archives of Dermatology Department).

**Figure 3:**
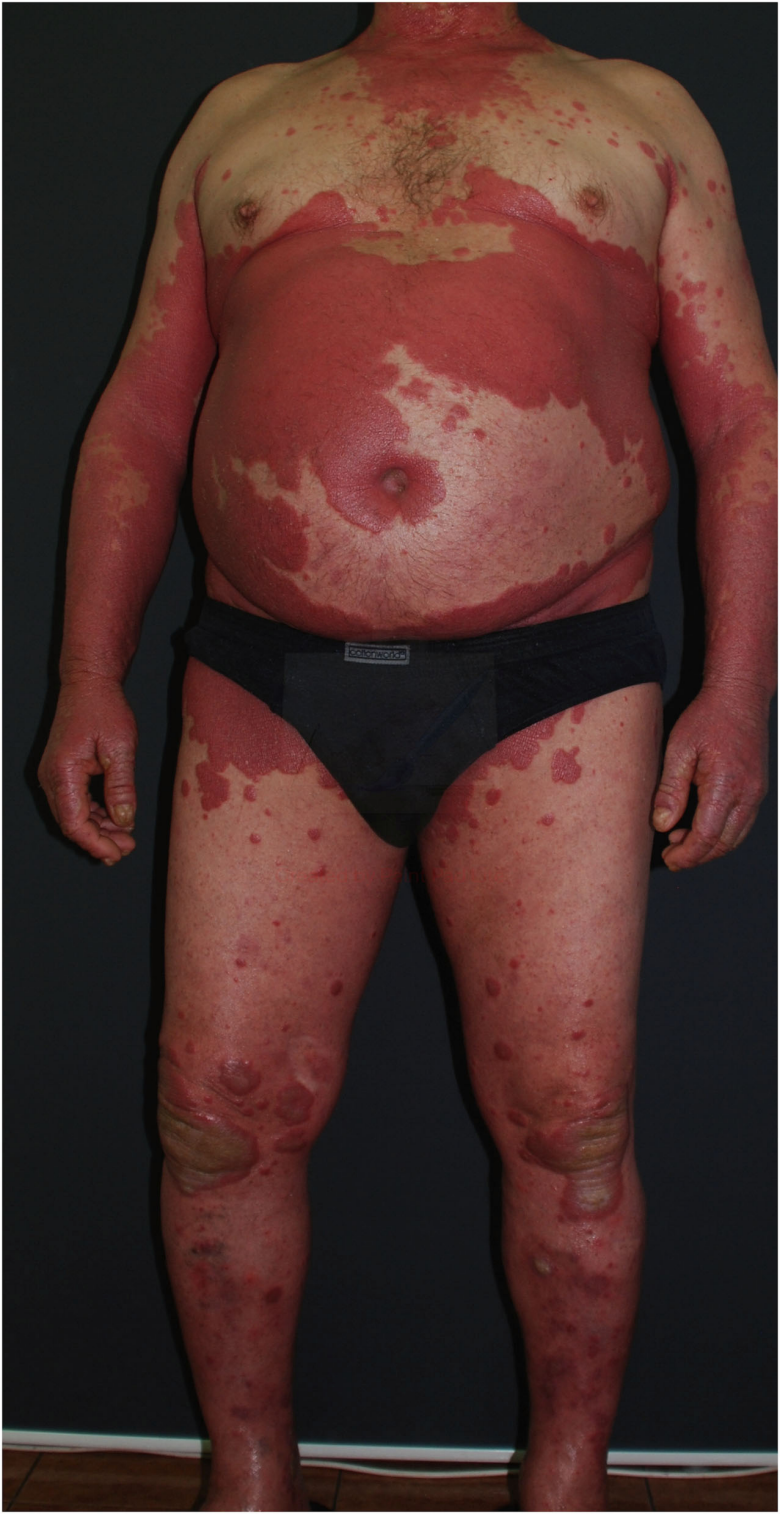
Erythematous and scaly psoriatic lesions affecting large area of the body (from the archives of Dermatology Department).

The most common type of psoriasis is plaque, which manifests as scaly papules and plaques, usually located on elbows, knees, and scalp, but which may appear in every body area (14) (Figures 2, 3).

Psoriasis may present with pruritus or burning sensations or, in the case of accompanying psoriatic arthritis, with joint pain, which can lead to difficulty with falling asleep or awakening (14). An impaired thermoregulation process is described in psoriatic patients, which may additionally interrupt sleeping (13). Psoriasis is well-documented to be associated with decreased levels of adiponectin (44). This happens to be a similar finding to what is observed after sleep deprivation (30). Moreover, sleep loss increases the risk of obesity and cardiovascular diseases (9). Therefore, sleep deprivation, which itself increases the risk of disorders observed in metabolic syndrome, can also aggravate such symptoms in patients with psoriasis. Studies on SD performed on mice with induced psoriasis reveal increased levels of pro-inflammatory cytokines, IL-1b, IL-6, and IL-12, and decreased levels of the anti-inflammatory cytokine IL-10 (14). Moreover, depression, which affects about 20–30% of psoriatic patients, has to be considered as an important factor causing sleep deprivation in this group (45, 46).

In our previous research, we proved that patients with psoriasis had worse sleep quality than people without skin diseases. We also found that such patients slept significantly fewer hours and took more sleeping medications. Therefore, we believed that SD should be taken into account in daily clinical practice, and reliable recommendations regarding its early detection should be established (6).

### The Influence of Psoriasis on Obstructive Sleep Apnea

Patients with psoriasis are also reported to have an increased risk of OSAS (7, 47, 48). This could be explained by their common relationships with metabolic disorders and molecular findings, such as inflammatory processes and oxidative stress (48, 49). Psoriasis is known to increase the risk of obesity—one of the main risk factors of OSAS (50). Some authors even suggest that psoriasis is an independent risk factor for OSAS development (51). In our previous research, we receive similar outcomes: Patients with psoriasis have increased risk of OSAS, and this risk increases with the duration of psoriasis (6). OSAS presents clinically with apneas, hypopneas, loud snoring, and awakenings (21). Therefore, it interrupts sleep course and contributes to insufficient sleep quality, which results in daytime sleepiness and fatigue. Psoriasis, tightly associated with metabolic disorders, may also even exacerbate the negative consequences of OSAS on the cardiovascular system: increased sympathetic activity, elevated blood pressure or endothelial dysfunction and, therefore, increased risk of arterial hypertension, coronary heart disease, arrhythmias, and stroke development (52, 53).

### The Influence of Psoriasis on RLS

A few papers also mention a possibly higher risk of RLS occurrence in the group of patients with psoriasis (47, 54). Among all SD in such patients, RLS is probably the least studied and uncertain. Nevertheless, in our previous research, we proved that patients with psoriasis had more severe symptoms of RLS (6). Similar outcomes are obtained by Saçmaci et al. (7). The possible connection between psoriasis and RLS may be the etiology, which is suspected to be autoimmune and inflammatory in both diseases and associations of both entities with metabolic disorders (6).

Considering the symptoms of RLS, such as the urge to move the legs and accompanying unpleasant sensations and the fact that they exacerbate at night, they interfere with sleep course causing difficulties falling asleep or awakening and lead to worse sleep quality in patients with psoriasis (55). Similar to OSAS, RLS indirectly causes disturbances in nocturnal rest, daytime dysfunction, and tiredness.

**Figure 4:**
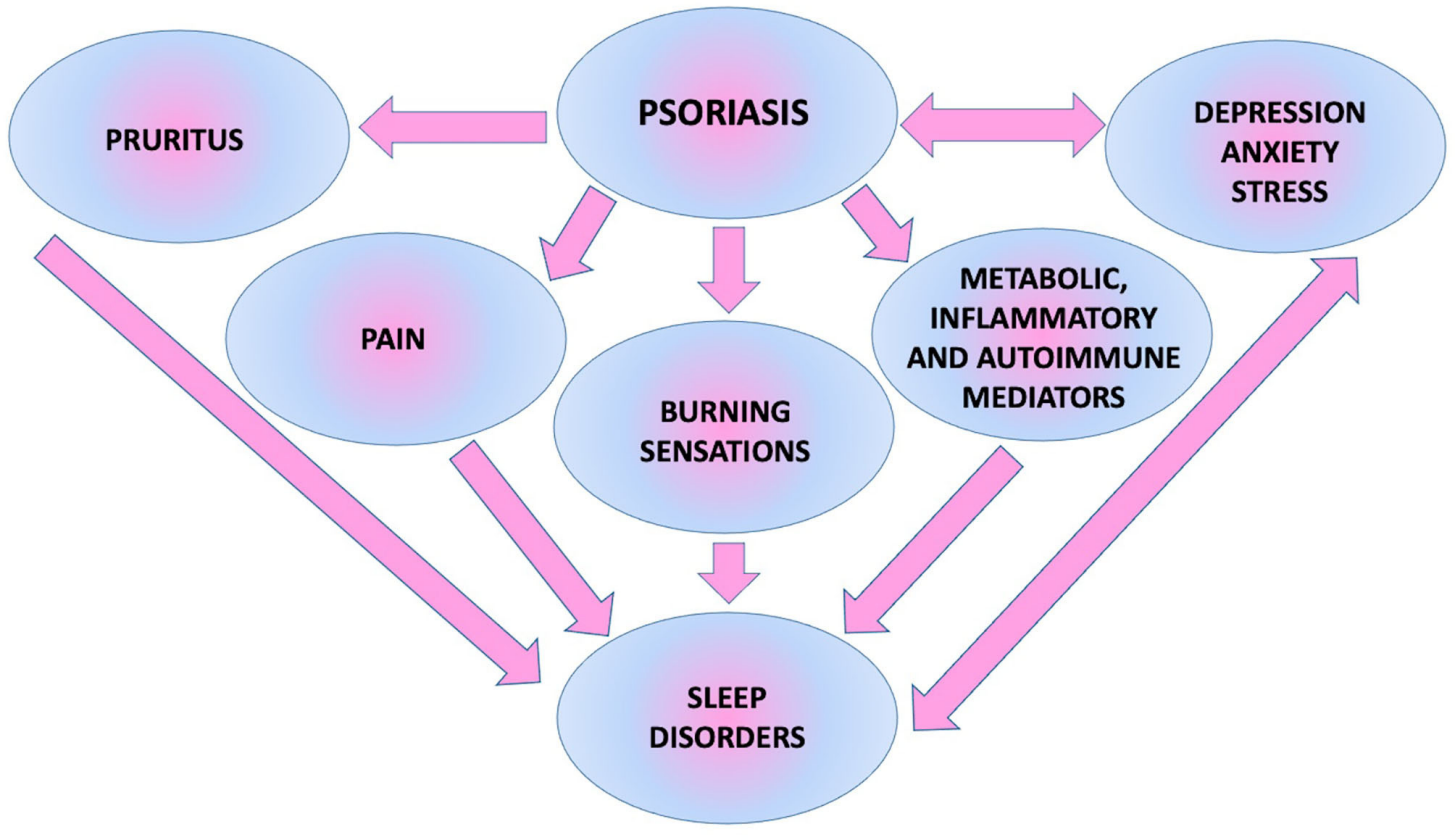
Factors associated with psoriasis which might negatively affect sleep course.

All possible factors influencing sleep in patients with psoriasis are presented in Figure 4 (6).

## Mutual Links Between Sleep Disorders in Psoriatic Patients and Their Quality of Life and Psychosocial Status

Psoriasis requires special attention because it is a great economic burden. It is all the more distressing because the global incidence of this dermatosis has been noted to increase within the past three decades (56). It is estimated that the total burden of psoriasis is 35.2 billion dollars, 12.2 billion dollars of which being incremental medical costs and 11.2 billion dollars as productivity losses (57). However, one must also take into account treatment and complications of multiple systemic comorbidities of psoriasis; therefore, those numbers must actually be bigger (57).

**Figure 5:**
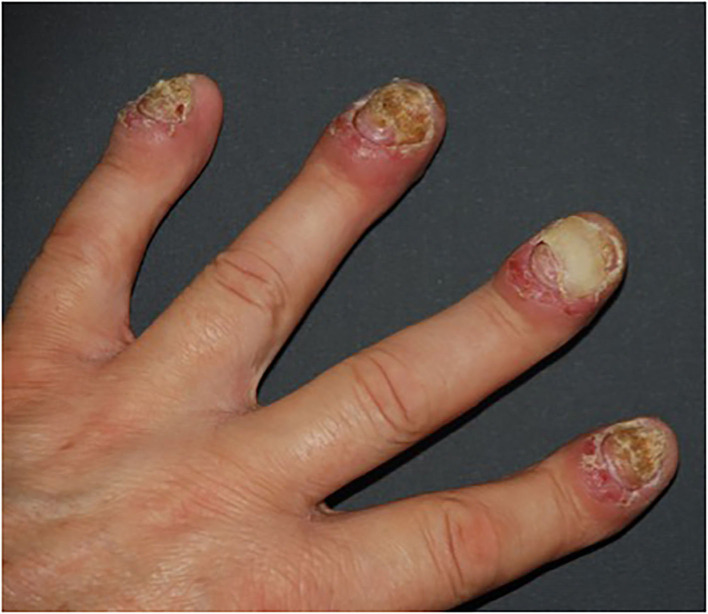
Severe, mutilating nail and finger lesions in acrodermatits continua of Hallopeau. Onycholysis, erythematous-oedematous lesions of distal parts of fingers (from own archives).

Patients with psoriasis, due to the skin lesions sometimes being extensive or accompanied by psoriatic arthritis, may not be able to take up employment; they cannot manage in everyday life (58). In other than the plaque type of psoriasis, for instance, acrodermatitis continua of Hallopeau or palmoplantar pustular psoriasis, sterile pustules on hands, fingertips, feet, and toes appear, and also nails can be involved (59) (Figure 5).

In generalized pustular psoriasis, painful and disfiguring skin lesions accompanied by sepsis-like systemic symptoms occur (60). These are examples of even more severe types of psoriasis, also leading to physical disability and incapability of task performance, which subsequently leads to absence at work (57, 58). Indeed, the involvement of palms, soles, and joints is reported to influence the number of absences significantly (58). Patients with psoriasis are reported to be less efficient workers (57). The National Psoriasis Association of America reports that such patients lose 56 million work hours every year (58). Research shows that severe psoriasis leads to occupational disability, which is an essential factor causing deterioration of the mental condition (8, 58). Patients with psoriasis may, therefore, develop feelings of frustration or fear of losing their employment (58). As described above, bad economic status results in stress and lower mood, which triggers SD.

Lack of employment and non-attendance at school or university may cause loss of social interactions. All of these factors taken altogether lead to social deterioration or the inability to start a family or cause the already existing family system to fall apart. It is well-reported that psoriatic patients have a lower ability to function in everyday life and create social bonds and have a disturbed sexual life (38, 61). The impact of psoriasis on everyday existence is dependent on sex and age. Women and younger patients seem to be more susceptible (8). In one study, the age group between 18 and 45 years presented trouble with daily activities and job and financial aspects as well as with social interactions and appearance acceptance (8, 62). Feelings that are frequently reported by patients with psoriasis and triggered by the dermatosis are stigma, shame, embarrassment, anger, frustration, and low self-esteem, which results in the abandoning of previous activities and social isolation (8). Patients who suffer from psoriasis are not confident about their physical appearance, and it seems that the feeling increases along with the severity of psoriasis from the patient's perspective (8). It leads to lower quality of life, an increased amount of stress, social stigmatization, and even psychological disorders and induction of SD (57).

Stress is a well-known factor contributing not only to exacerbation of psoriatic skin lesions, but also even triggering the onset of the disease (56). The impact of stressful events in triggering the guttatae type of psoriasis is reported to be about 1.7% (0.8–3.6) (8, 63). Therefore, stressful events are an important issue in this group of patients, and stress reduction must be a priority in order to improve both the mental and physical status of patients with psoriasis. Unaesthetic appearance of skin lesions further exacerbates stress, and the latter triggers sleep disorders.

The whole matter also has to be perceived from the other side. Patients with psoriasis, due to multiple factors and different pathogenic paths, perhaps some of them not yet discovered or defined, clearly suffer more often from SD, which additionally decreases their quality of life and increases the possibility of psychological disorders and addiction propensity (14, 45). Apart from the disease itself, which prevents patients from functioning normally in everyday life, insomnia and sleep loss due to OSAS or RLS resulting in daytime sleepiness additionally makes it even more difficult to perform a job or other duties. Sleep loss is reported to have a negative impact on social interactions due to lower mood, stress, irritation, and lower sex drive, all of which can be already affected by lower self-esteem and bad perceptions of one's physical appearance and attraction (24). What should be mentioned is that social pain has an impact not only on social behaviors, but interestingly also on the experience of physical pain, which accompanies some patients with psoriasis anyway (43). Psychiatric disorders are an important factor increasing annual direct healthcare costs in psoriatic patients, most commonly depression and anxiety disorders (57).

Sleep deprivation is also proved to cause numerous health problems and comorbidities. In patients who suffer from psoriasis, it is even more disturbing, seeing that psoriasis itself, as a disease of multifactorial pathogenesis, is known to be accompanied by many different illnesses. Patients who present at least one accompanying disease have higher rate of hospitalizations and appointments in ambulatory care (57). SD should be, therefore, perceived as an additional risk factor for psoriasis' wide comorbidity and independently increases the severity of accompanying illnesses.

A rarely raised but undoubtedly essential issue is that patients with psoriasis may have decreased fertility. It could be not only due to the impact of immune and inflammatory factors of this disease itself, along with its comorbidities (e.g., polycystic ovary syndrome is quite common in such patients), but also due to other causes: administered treatment (during the therapy with many classic antipsoriatic agents, pregnancy is contraindicated), improper lifestyle of psoriatic women, or negative effect of sleep deprivation (31, 64). Lower birthrates in this particular group of patients may become another medical and social issue, and deprived sleep could be one of the possible reasons (64).

Another problem in patients with psoriasis, who already have an increased risk of obesity, is the effect of stress and sleep deprivation on poor eating habits (24, 44, 50). First, diet is known to have an undoubtedly essential role in psoriasis. Studies report poor eating habits among patients with psoriasis who eat high-calorie foods with low content of proteins, complex carbohydrates, and fiber. Instead, they choose products containing simple sugars and fats. There are also reports that a low-energy diet can contribute to reduced severity of skin lesions in patients with psoriasis (44). There is a large body of evidence that chronic stress, which activates the HPA axis, is involved in the nutritional processes due to exacerbating stress eating. Activation of the HPA axis increases cortisol and ghrelin secretion, which next results in hunger, particularly for food providing better well-being. Then, the consumption of such products releases endogenous opioids, which improves the mood (24). This is a situation that is supposedly very common among patients with psoriasis because chronic stress is another factor contributing to their poor eating habits and obesity. Chronic stress can trigger sleep disturbances and vice versa; SD can contribute to excessive stress. Nevertheless, independently, individuals who suffer from insufficient sleep are more likely to present wrong eating habits (24). These are, for instance, intake of too high-calorie products containing large amounts of sugar, fat, and salt (24). Apparently, there are alterations in secretions of hormones that are involved in regulation of hunger-satiety sensation (24). Inappropriate nutrition, along with insufficient physical activity, which may also be lower due to sleep deprivation, contributes additionally to increased body mass in patients with psoriasis, already predisposed to obesity by the disease itself (44).

**Figure 6:**
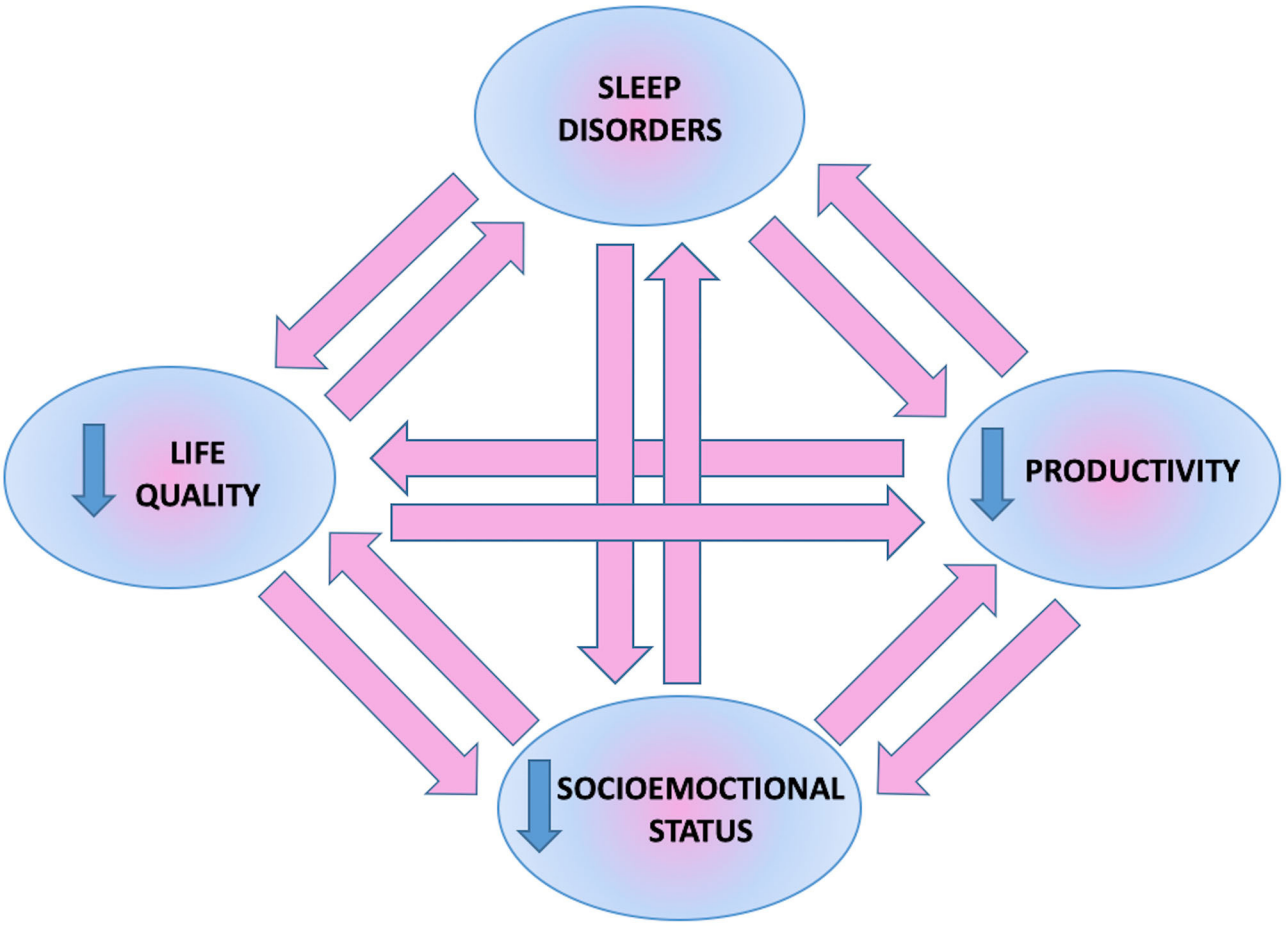
Mutual relationship between sleep disorders, psychoemotional and socioeconomic status of patients.

Mutual relationships between sleep disorders, quality of life, and psychological or economic aspects in psoriatic patients described above are presented in Figure 6.

Measures should be taken in order to stop this vicious circle. This could be achieved by a few simple actions. First of all, most important is the early diagnosis of psoriasis, followed by introduction of an adequate treatment. Although the severity of psoriatic lesions in the Psoriasis Activity and Severity index is the most influencing factor affecting choice of drug, others also have to be taken into account, such as the localization of lesions and the quality of life along with the impact of psoriasis on everyday existence (65). The proper analysis of drug choice is also complex in regard to the total costs of treatment. Not only should the cost of a single pack be taken into account, but also the amount of drug that has to be applied in a defined period of time as well as its efficacy. Sometimes, despite the high price of a single pack of a particular drug compared with other medications, it could be more efficient and, therefore, used for a shorter period of time or in a smaller amount (66). What is more, different therapeutic methods are associated with diverse screening and monitoring tests or are time-consuming (67). These issues should also be carefully analyzed because they affect the total costs of treatment, including inability to work and earn money. A second activity is proper education of patients. The thorough explanation of treatment along with non-pharmacological actions affecting psoriatic skin is crucial so that the patient is actually adherent to the doctor's advice. Moreover, patients with psoriasis should be encouraged to attend psychologists and/or psychiatrists in order to get help for their mental problems. Mild psychosocial disturbances with decreased quality of life could be sufficiently managed by a psychologist, for instance, with psychotherapy. More severe disorders, especially SD and also depression or anxiety should be consulted and treated by psychiatrists.

## Conclusions

Psoriasis is one of the most frequent skin diseases in dermatological practice. It is also one of the most common reasons for hospital admission to dermatology departments. Moreover, psoriasis is associated with numerous comorbidities, especially with cardiovascular disorders, which are the first cause of death in the world. This skin disease also leads to lower productivity of such patients, greater rates of absence at work, and lower ability to learn and study. Considering all the above factors, psoriasis is an essential and current medical as well as social issue and requires special attention and still more in-depth medical investigations to fully understand its nature and help patients in everyday life. SD, although intangible and invisible on physical examination, are a real medical problem, which has numerous serious negative consequences of a psychological and somatic nature. The relationship between SD and psychosocial status is bidirectional and resembles a vicious circle, one abnormality triggering the other. SD additionally increase the risk of metabolic and psychiatric diseases in psoriatic patients, who already have this possibility increased. These complex dependencies should be, therefore, thoroughly understood by physicians in order to provide a holistic approach to their patients.

## Author Contributions

JN: conceptualization, data curation, investigation, visualization, project administration, resources, writing–original draft, and writing–review and editing. AB: conceptualization, data curation, project administration, writing–review and editing, supervision, and funding acquisition. IF: project administration and supervision. All authors contributed to the article and approved the submitted version.

## Conflict of Interest

The authors declare that the research was conducted in the absence of any commercial or financial relationships that could be construed as a potential conflict of interest.
